# Supplementary material for: Mitochondrial reactive oxygen species regulate the induction of CD8+ T cells by plasmacytoid dendritic cells
Source: Nat Commun. 2018 Jun 8;9:2241. doi: 10.1038/s41467-018-04686-8 (PMC5993805; doi:10.1038/s41467-018-04686-8)
Supplement: Supplementary file 1 — Supplementary Information [file 41467_2018_4686_MOESM1_ESM.pdf]

SUPPLEMENTARY INFORMATION

Mitochondrial reactive oxygen species regulate the induction of CD8$^+$ T cells by plasmacytoid dendritic cells

Oberkampf et al.

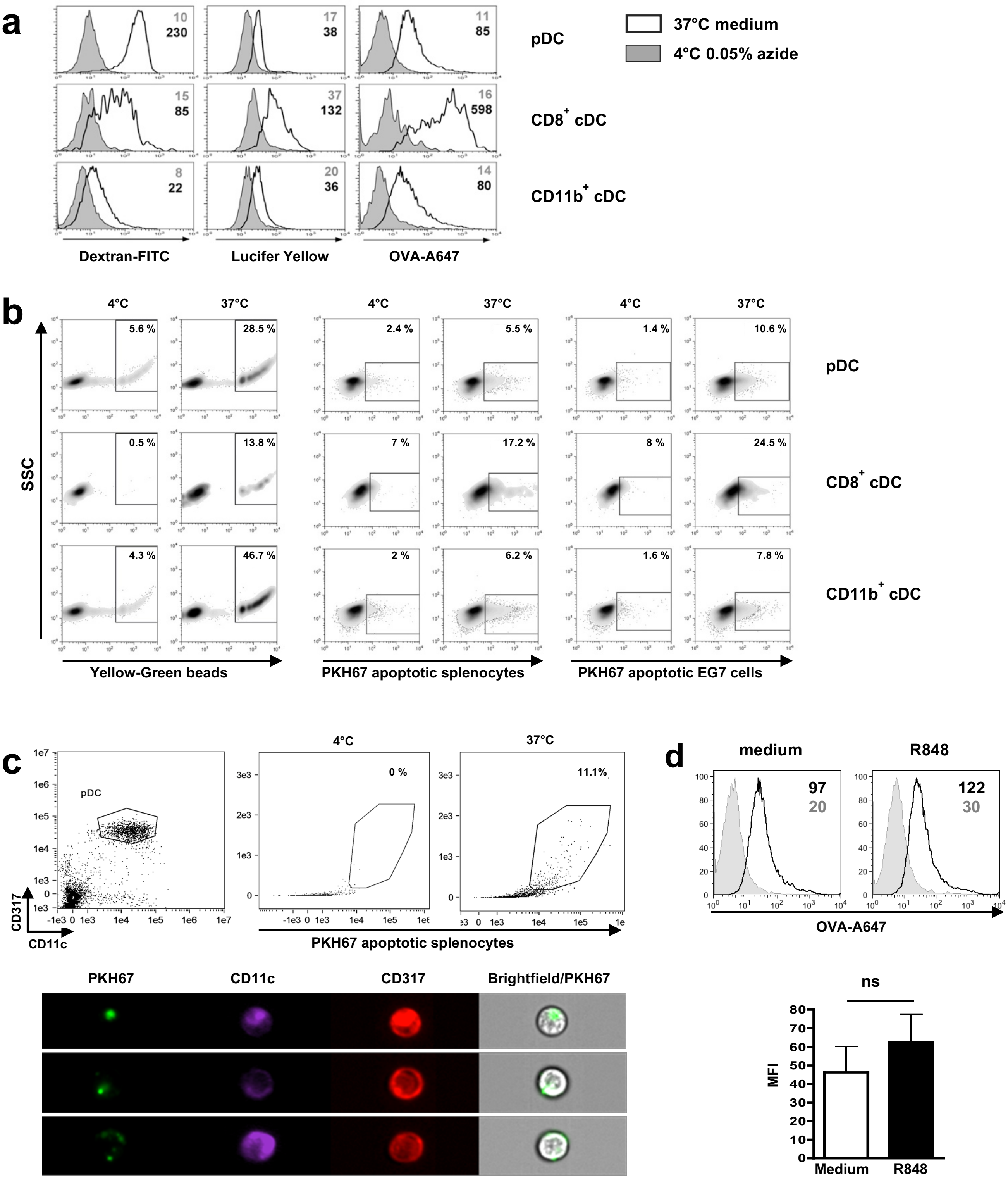
a
37°C medium
4°C 0.05% azide
pDC
CD8+ cDC
CD11b+ cDC
10
230
17
38
11
85
15
85
37
132
16
598
8
22
20
36
14
80
Dextran-FITC
Lucifer Yellow
OVA-A647
b
4°C
37°C
SSC
5.6 %
28.5 %
2.4 %
5.5 %
1.4 %
10.6 %
0.5 %
13.8 %
7 %
17.2 %
8 %
24.5 %
4.3 %
46.7 %
2 %
6.2 %
1.6 %
7.8 %
Yellow-Green beads
PKH67 apoptotic splenocytes
PKH67 apoptotic EG7 cells
c
pDC
CD317
CD11c
0 %
11.1%
PKH67
CD11c
CD317
Brightfield/PKH67
d
medium
R848
97
20
122
30
OVA-A647
ns
MFI
Medium
R848

Supplementary Figure 1. Resting pDCs efficiently capture Ags. (a) The DC subsets were incubated for 30 min in the presence of labeled Ags (Dextran-FITC (0.5 mg/mL), Lucifer Yellow (0.5 mg/mL) or OVA-A647 (0.5 mg/mL)), and the cells were labeled and analyzed by flow cytometry. The panels show the fluorescent intensity of the labeled Ags analyzed for pDCs, $CD8^+$ cDCs and $CD11b^+$ cDCs incubated at 37° C (thick line histogram) or 4° C in a medium containing 0.05 % azide (filled histogram). MFI are indicated in the corner of each histogram (black: 37°C; gray: 4°C). **(b)** The DC subsets were incubated for 30 min at 37°C in the medium alone or 4°C in a medium containing 0.05 % azide in the presence of Yellow-Green beads ($5x10^8$ beads/mL), PKH67-labeled apoptotic splenocytes ($2x10^6$ per well) or PKH67-labeled apoptotic EG7 cells ($2x10^6$ per well). The panels show the Ag internalization, gated on pDCs, $CD8^+$ cDCs and $CD11b^+$ cDCs. The numbers indicate the percentage of DCs with internalized Ags. One representative experiment of 4 is depicted. **(c)** pDCs were incubated for 30 min at 37°C in medium alone or at 4°C in medium containing 0.05 % azide, in the presence PKH67-labeled apoptotic splenocytes ($2x10^6$ per well). The top panels show the Ag internalization, gated on pDCs. The numbers indicate the percentage of pDCs with internalized Ags. The bottom panel shows morphology and fluoresence by imaging. One representative experiment of 2 is depicted. **(d)** pDCs were incubated for 30 min in the presence of OVA-A647 (0.5 mg/mL) at 37°C in the medium alone or 4°C in a medium containing 0.05 % azide with or without R848 (1 μg/mL). The fluorescent intensity of OVA-A647, gated on pDCs ($CD11c^{low}$ $CD11b^-PDCA1^+$), is shown after an incubation at 37° C (thick line histogram) or 4° C (filled histogram). The top panel shows histograms of one representative experiment. The numbers represent the MFI obtained at 37°C in the medium (black) or 4°C in a medium with 0.05 % azide (gray). The bottom panel depicts the mean MFI (MFI at 37°C - MFI at 4°C) ± SD obtained from 3 independent experiments. ns: non-significant, paired t-test.

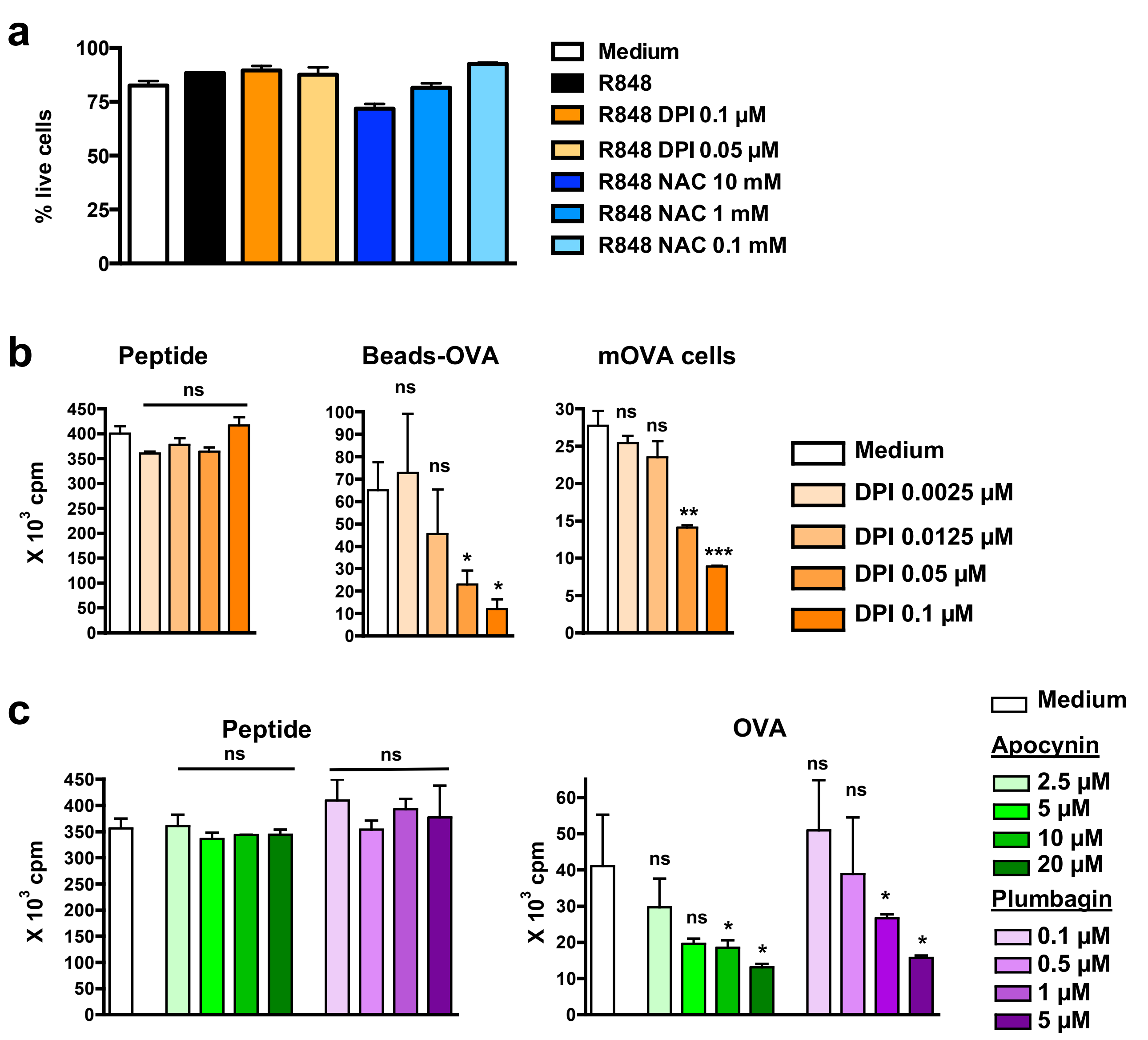

Supplementary Figure 2. (a) Purified pDCs from C57BL/6 mice were incubated for 1 hour at 37°C in the medium with or without R848 and/or DPI or NAC at the indicated concentrations. Then, the cells were stained with the CD11c and PDCA1 antibodies and DAPI. The data indicate the percentage of live cells (DAPI$^-$) among the pDCs (CD11c^{low}PDCA1$^+$). One representative experiment of 3 is depicted. **(b)** Purified pDCs from C57BL/6 mice were incubated in the presence of R848 (1 µg/mL) and the SIINFEKL peptide (0.001 µg/mL), beads-OVA (10^7 beads/mL) or apoptotic Act-mOVA/ Kb$^{-/-}$ splenocytes (mOVA cells, 10^5 cells/well) with serial dilutions of DPI. **(c)** Purified pDCs from C57BL/6 mice were incubated in the presence of R848 (1 µg/mL) and the SIINFEKL peptide (0.001 µg/mL) or the OVA protein (1 mg/mL) and serial dilutions of apocynin or plumbagin. **(b, c)** After washing, the pDCs were co-cultured with LN cells from OT-I Rag$^{-/-}$ mice for 72 hrs, and [^{3}H]-thymidine was added for the last 18 hours of culture. The T cell proliferation is expressed as the mean cpm ± SD of triplicates. One representative experiment of 2 is depicted. Significant differences were analyzed for each condition compared to the medium alone with an unpaired t-test: ns: non-significant, *p<0.05; **p<0.01; ***p<0.001.

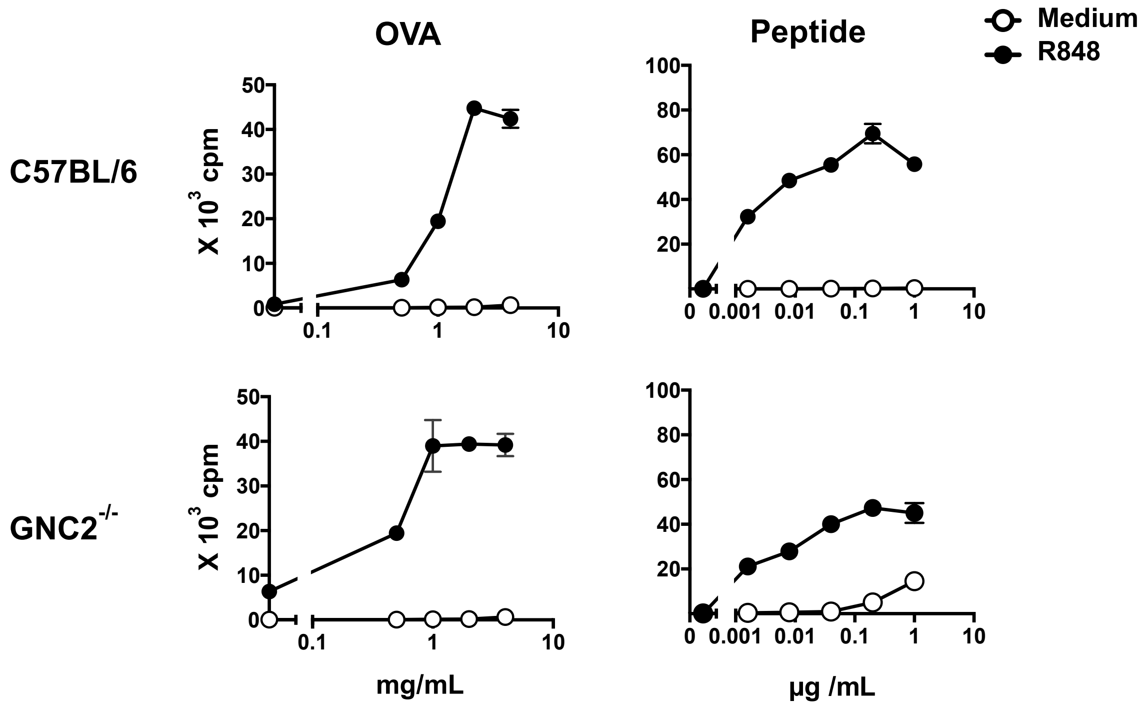

Supplementary Figure 3. Induction of cross-presentation by pDCs is independent of GCN2 and autophagy. Purified pDCs from C57BL/6 or GNC2$^{-/-}$ (B6.129S6-*Eif2ak4*$^{tm1.2Dron}$/J autophagy-deficient mice (Ravindran et al, 2014, Science, 343, 313-317) from Jackson laboratory) were incubated for 1 hour at 37°C in the medium alone or with R848 (1 µg/mL) and serial dilutions of OVA or the SIINFEKL OVA peptide. After washing, the pDCs were co-cultured with LN cells from OT-I Rag$^{-/-}$ mice for 72 hours, and [^{3}H]-thymidine was added for the last 18 hours of culture. The T cell proliferation is expressed as the mean cpm ± SD of triplicates. One representative experiment of 2 is depicted.

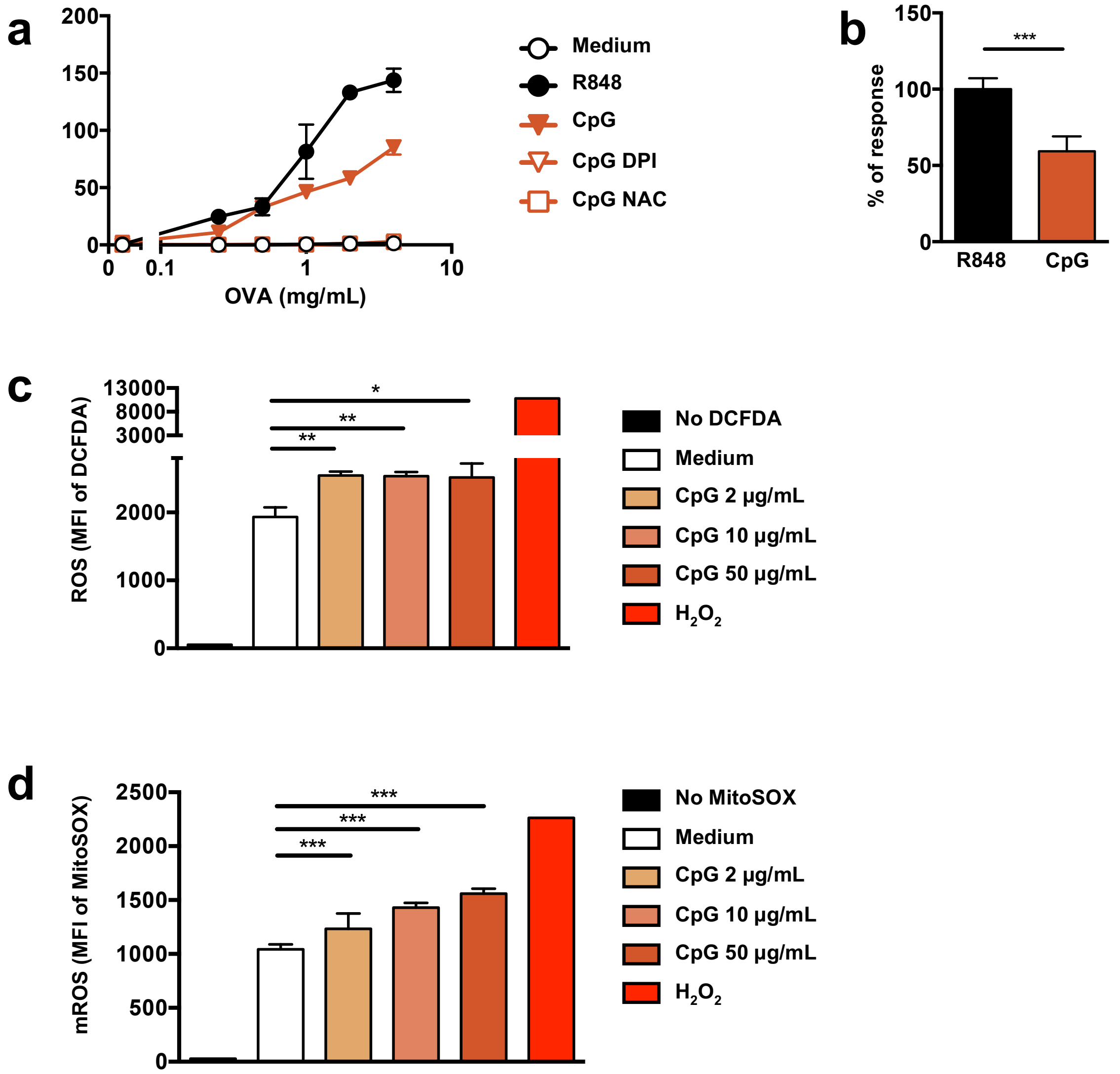

Supplementary Figure 4. The activation of pDCs by CpG induces cross-presentation and mROS production. (a, b) Purified pDCs were incubated with serial dilutions of OVA in medium with CpG alone or with DPI (0.1 µg/mL) or NAC (10 mM). After washing, pDCs were co-cultured for 72 hours with LN cells from OT-I Rag$^{-/-}$ mice. **(a)** The T cell proliferation is expressed in cpm of incorporated [^{3}H]-thymidine. The results are shown as the mean cpm ± SD of triplicates. One representative experiment of 3 is depicted. **(b)** The results show the cumulative data from 3 experiments using CpG-activated pDCs from C57BL/6 loaded with the OVA protein (4 mg/mL) in the absence of inhibitor. Data are expressed as the mean ± SD percentage of the response obtained with C57BL/6 pDCs. **(c)** The production of ROS in the cytosol of C57BL/6 pDCs was measured by the quantification of the DCFDA fluorescence by flow cytometry. pDCs purified from C57/BL/6 mice were loaded with DCFDA and then activated with serial concentrations of CpG for 30 min. **(d)** The production of mROS by pDCs was measured by the quantification of the MitoSOX fluorescence by flow cytometry. pDCs purified from C57BL/6 mice were activated by serial concentrations of CpG for 30 min and then loaded with MitoSOX. Results show the cumulative results from triplicates and are representative of 2 independent experiments. Significant differences were analyzed for each condition with an unpaired t-test: *p<0.05; **p<0.01; ***p<0.001.

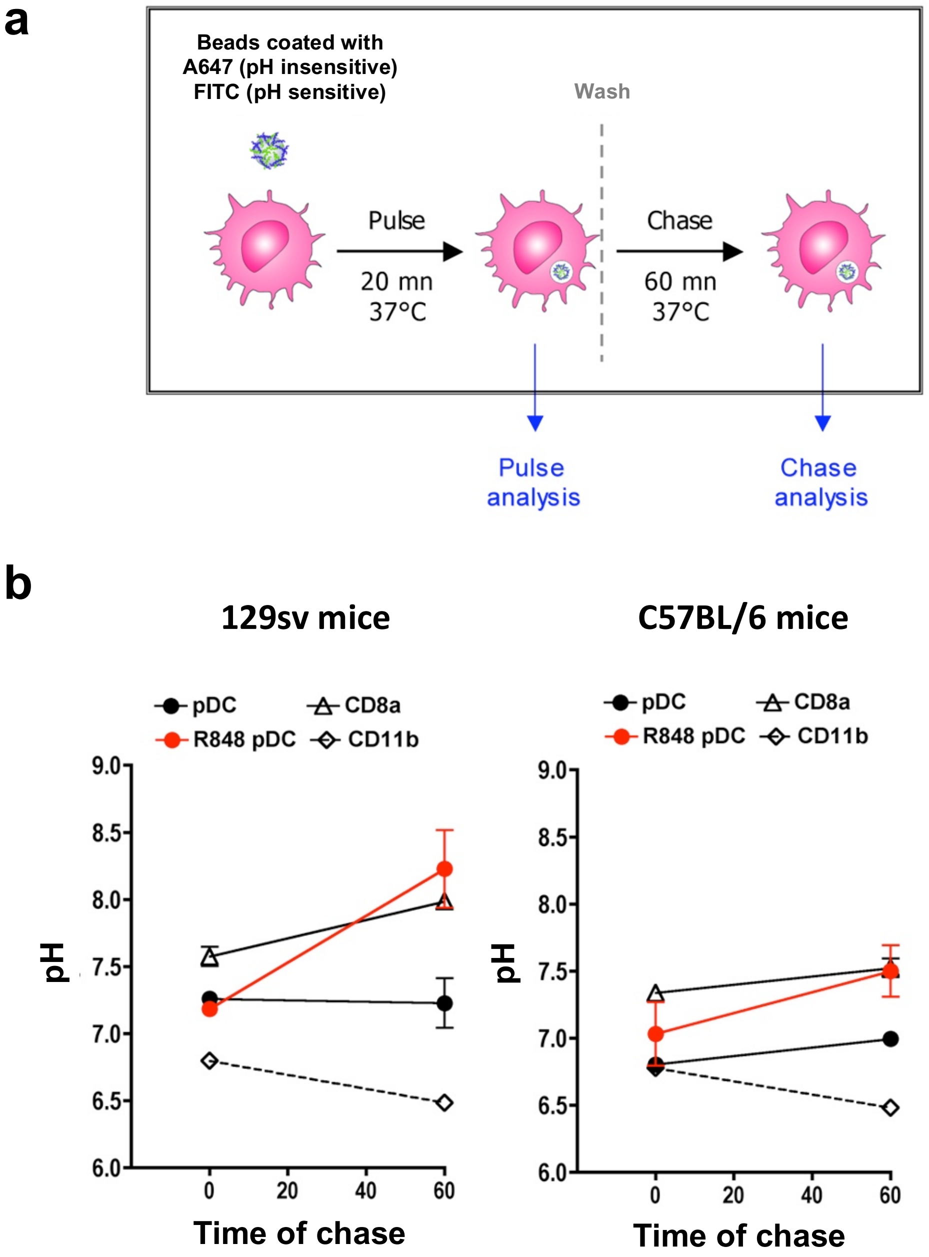

Supplementary Figure 5. Phagosomal pH in the DCs populations. (a) Protocol of pH assay. cDCs and pDCs were pulsed with beads covalently linked with FITC (pH sensitive) and A647 (pH insensitive) for 20 min at 37°C. After washing, the cells were resuspended in the medium and either kept on ice (“Pulse”) or incubated at 37°C for 1 hour (“Chase”). The ratio of MFI between the two dyes was determined by FACS analysis. The pH values were determined with a standard curve obtained by suspending the cells in the medium at different fixed pH levels. **(b)** The pDCs and cDC subsets from 129/sv and C57BL/6 mice were purified and submitted to the pH assay. The pDCs were either untreated or activated with R848 (1 µg/mL) for 30 min before the assay. The results are expressed as the mean pH ± SD of triplicates. One representative experiment of 2 is depicted.

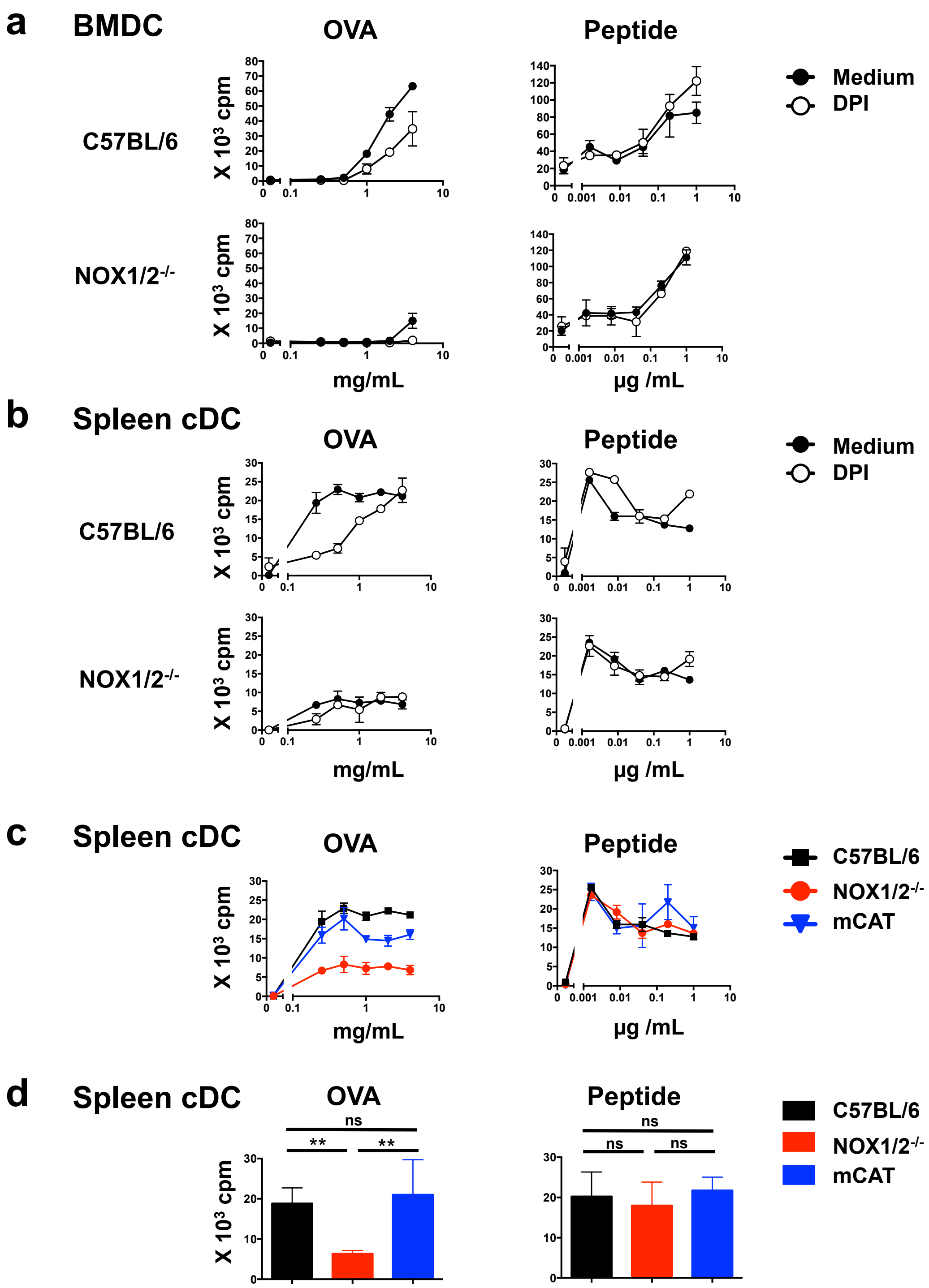

a
BMDC
OVA
Peptide
C57BL/6
NOX1/2-/-
X 10³ cpm
Medium
DPI
mg/mL
µg /mL
b
Spleen cDC
OVA
Peptide
C57BL/6
NOX1/2-/-
X 10³ cpm
Medium
DPI
mg/mL
µg /mL
c
Spleen cDC
OVA
Peptide
X 10³ cpm
C57BL/6
NOX1/2-/-
mCAT
mg/mL
µg /mL
d
Spleen cDC
OVA
Peptide
ns
**
**
ns
ns
ns
X 10³ cpm
C57BL/6
NOX1/2-/-
mCAT

Supplementary Figure 6. Cross-presentation by cDCs is dependent on NOX1/2. (a) BMDC were generated by culturing bone marrow cells from C57BL/6 or NOX1/2$^{-/-}$ mice in the presence of GM-CSF for 6 days. Then, the BMDC were incubated with serial dilutions of OVA or the SIINFEKL OVA peptide in the medium alone or with DPI (0.1 μg/mL) for 1 hour. After washing, the BMDC were cultured with LN cells from OT-I Rag$^{-/-}$ mice for 72 hours, and [^{3}H]-thymidine was added for the last 18 hours of culture. The T cell proliferation is expressed as the mean cpm ± SD of triplicates. One representative experiment of 2 is shown. **(b)** CD11c$^+$ cDCs purified from the spleens of C57BL/6 or NOX1/2$^{-/-}$ mice were incubated with serial dilutions of OVA or the SIINFEKL OVA peptide in the medium alone or with DPI (0.1 μg/mL) for 1 hour. After washing, the cDCs were cultured with LN cells from OT-I Rag$^{-/-}$ mice for 72 hours, and [^{3}H]-thymidine was added for the last 18 hours of culture. The T cell proliferation is expressed as the mean cpm ± SD of triplicates. One representative experiment of 3 is shown. **(c)** cDCs purified from the spleens of C57BL/6**,** NOX1/2$^{-/-}$ or mCAT mice were incubated with serial dilutions of OVA or the SIINFEKL OVA peptide for one hour. After washing, the cDCs were co-cultured with LN cells from OT-I Rag$^{-/-}$ mice for 72 hours, and [^{3}H]-thymidine was added for the last 18 hours of culture. The T cell proliferation is expressed as the mean ± SD of cpm of triplicates. One representative experiment of 2 is shown. **(d)** The results show the cumulative data of 2 experiments using cDCs purified from the spleens of C57BL/6, NOX1/2$^{-/-}$ or mCAT mice, loaded with the OVA protein (4 mg/mL) or the SIINFEKL OVA peptide (4 μg/mL) in the absence of DPI. Data are expressed as the mean ± SD percentage of the response obtained with C57BL/6 pDCs. Significant differences were analyzed for each condition with an unpaired t-test: ns: non significant; **p<0.01.

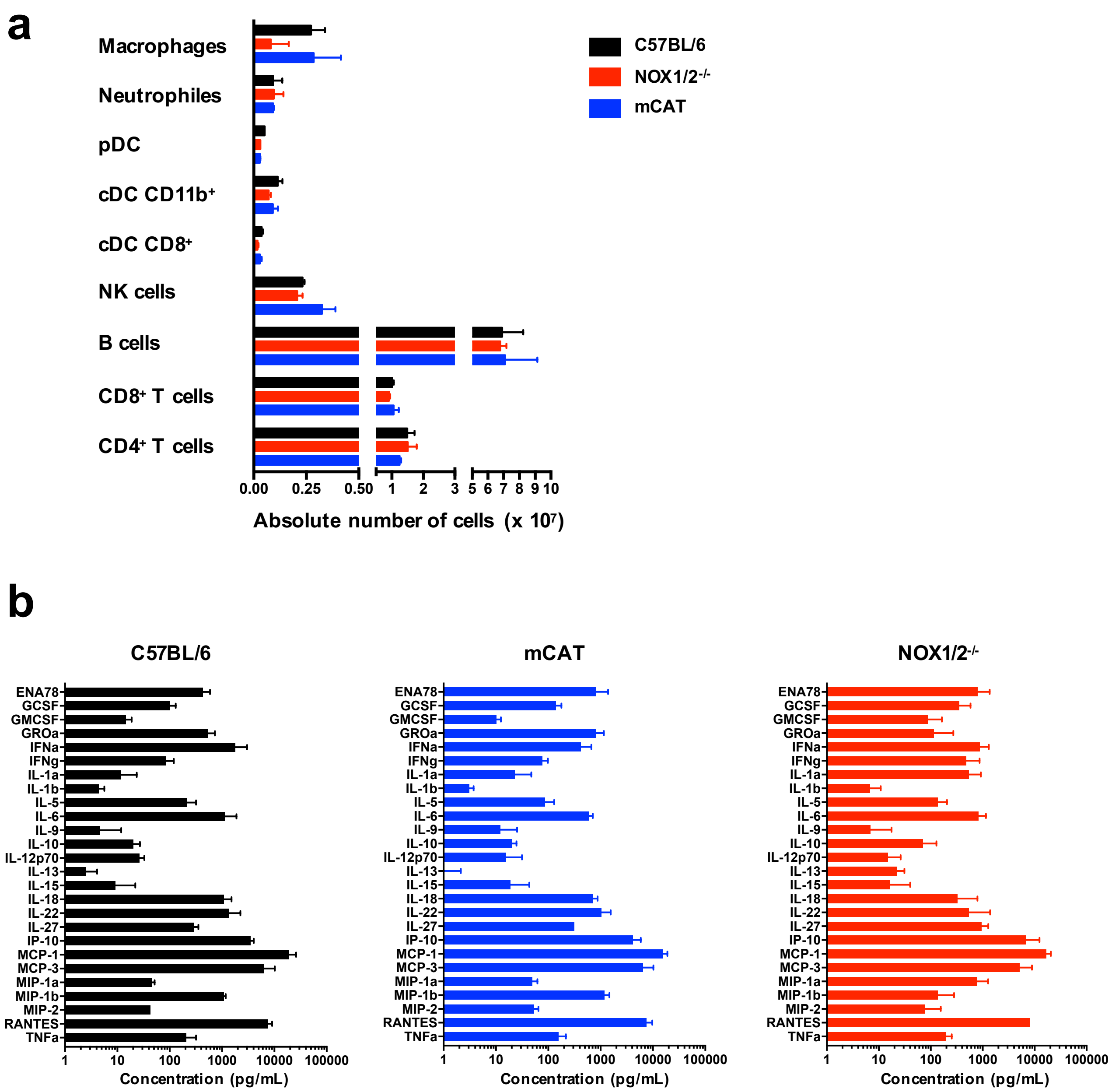

Supplementary Figure 7. The low CD8$^+$ T cell responses triggered in mCAT are not due to intrinsic defect of T cells or of innate immune responses. (a) Immune cells from the spleens of C57BL/6, mCAT and NOX1/2$^{-/-}$ mice were characterized by flow cytometry. Cell populations were defined as follow: Macrophages (CD11b$^+$ F4/80$^+$), Neutrophiles (CD11b$^+$ Gr1$^+$), pDCs (CD11c^{low} B220$^+$ CD317$^+$), cDCs (CD11c^{high} CD11b$^+$ or CD11c^{high} CD8$^+$), NK cells (CD3$^-$ NK1.1$^+$), B cells (CD3$^-$ CD19$^+$), T cells (CD19$^-$ CD3$^+$). **(b)** C57BL/6, mCAT and NOX1/2$^{-/-}$ were immunized with 30 µg of CpG/DOTAP. After 4 hours, blood samples were collected. Cytokine and chemokine concentration in the serum was assessed with the 36-plex Luminex immunoassay. IL-2, IL-3, IL-4, IL-17a, IL-23, IL-28, IL-31, LIF, MCSF and Eotaxin are not presented on the figure due to their low (≤ 10 pg/mL) or non-detectable expression in the samples. Data are expressed as the mean concentration ± SD of 4 mice per group in two distinct experiments.

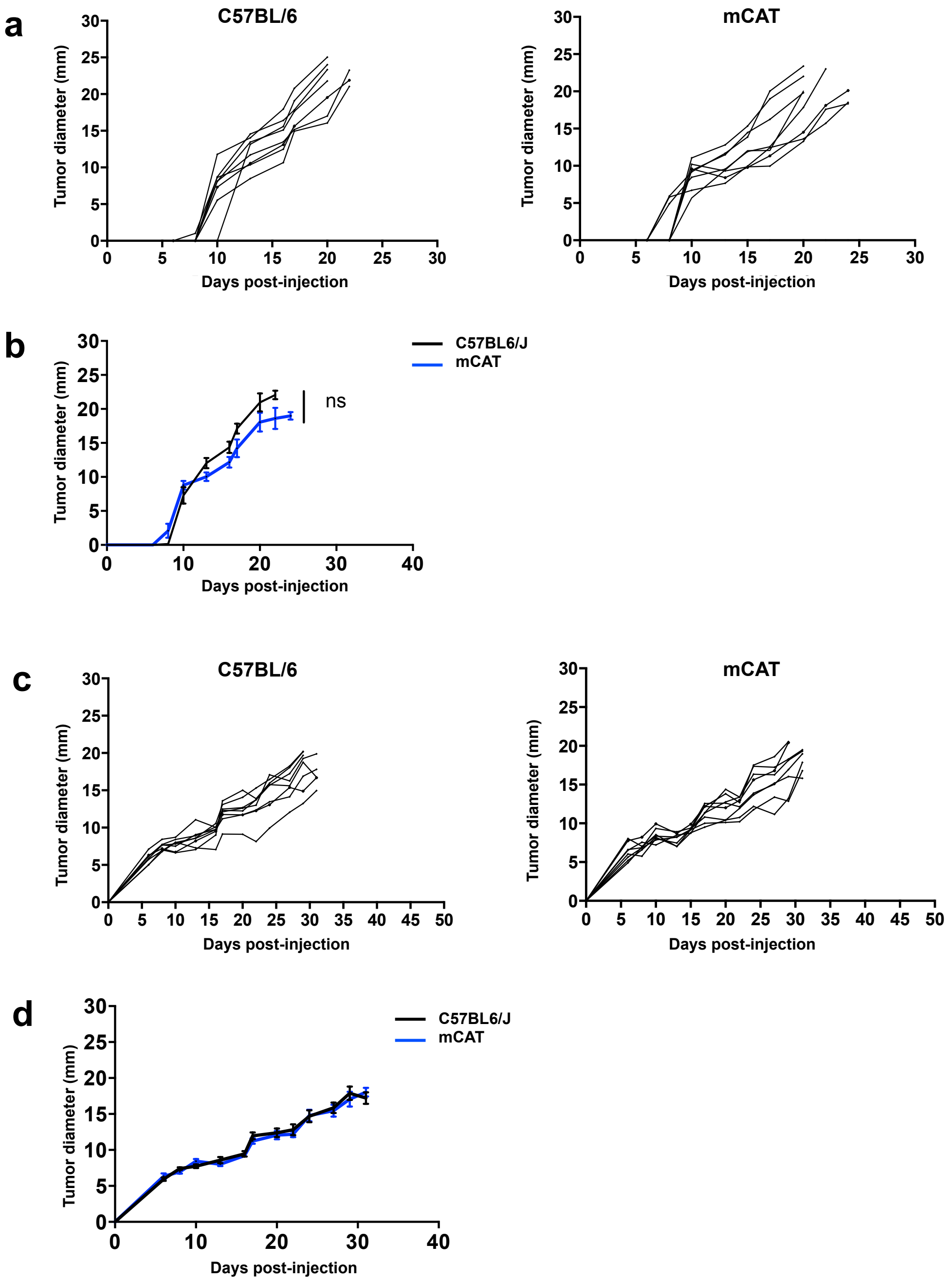

Supplementary Figure 8. **The reduced production of mROS in mCAT mice does not affect their resistance to tumor.** C57BL/6 and mCAT mice (n=8 per group) were grafted with 2.5 x 10^5 B16-OVA **(a-b)** or 6 x 10^5 TC-1 **(c-d)** tumor cells on day 0 and the tumor growth was followed until the tumor diameter reached 20 mm. **(a, c)** Each curve represents the tumor diameter of a single mouse. **(b, d)** The curves represent the mean ± SD of the tumor diameter for each group of mice. Unpaired t-test: ns: non-significant.

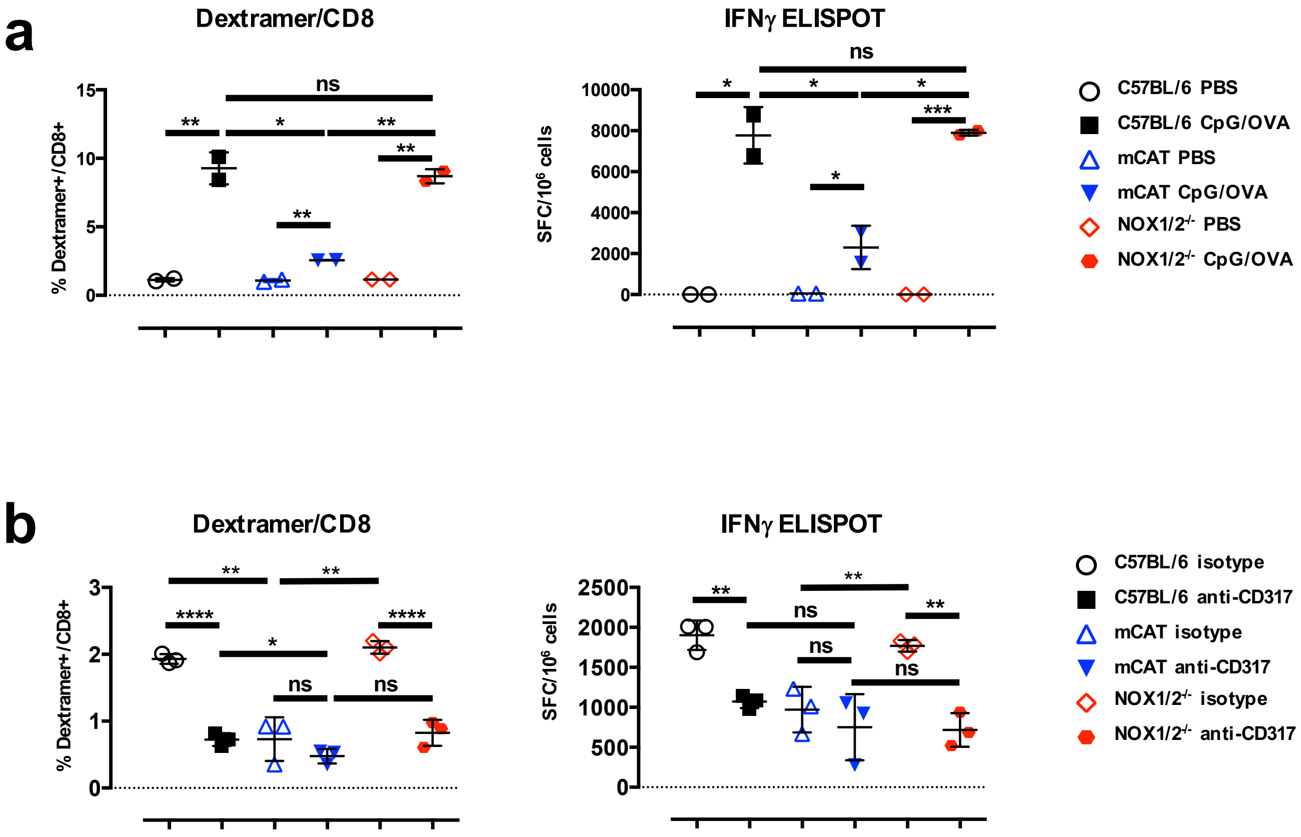

Supplementary Figure 9. pDCs play a crucial role in the induction of $CD8^+$ T cell responses following immunization by OVA/CpG. (a) 5.10^5 CFSE-labelled OT-I $CD8^+$ T cells were injected to C57BL/6, mCAT and $NOX1/2^{-/-}$ mice. One day later, mice were immunized with 100 µg of OVA mixed with 30 µg CpG/DOTAP. Four days later, the anti-OVA $CD8^+$ T cell response was assessed by SIINFEKL/H2-K^b dextramer staining and IFN-γ ELISPOT. **(b)** C57BL/6, mCAT and $NOX1/2^{-/-}$ mice were treated with anti-CD317 mAb or isotype control on days -1, +1 and +4 to deplete pDCs. The mice were immunized on day 0 with 100 µg of OVA mixed with 30 µg CpG/DOTAP. Seven days after immunization, the anti-OVA $CD8^+$ T cell response was assessed by SIINFEKL/H2-K^b dextramer staining and IFN-γ ELISPOT. (**a, b**) The results are expressed as the percentage of SIINFEKL/H-2K^b $dextramer^+$ among total $CD8^+$ splenocytes for dextramer staining and IFN-γ spot-forming cells (SFC) per 10^6 splenocytes for ELISPOT. Unpaired t-test: ns: non-significant, *$p<0.05$; **$p<0.01$; ***$p<0.001$; ****$p<0.0001$

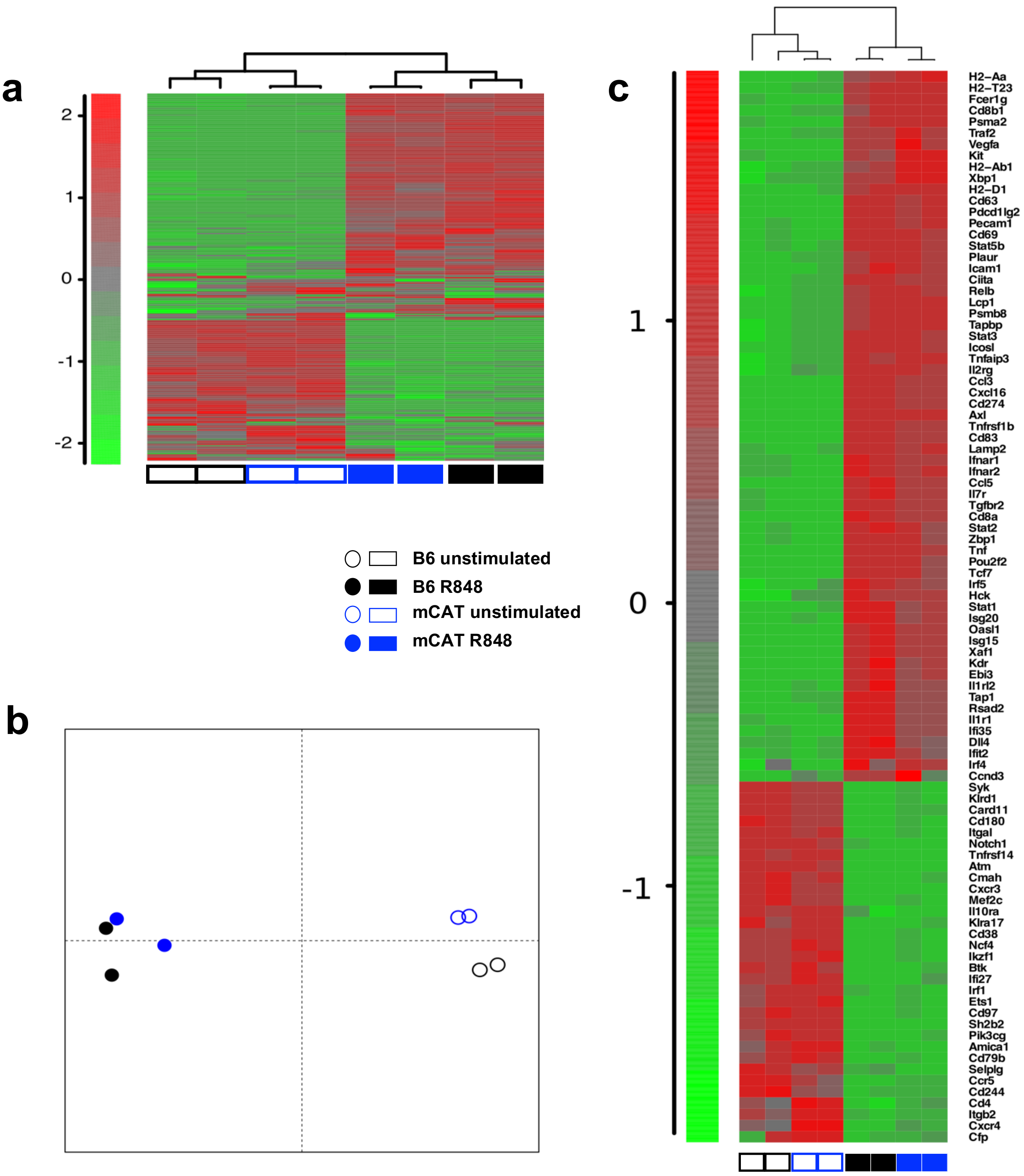
a
2
1
0
-1
-2
b
c
1
0
-1
B6 unstimulated
B6 R848
mCAT unstimulated
mCAT R848
H2-Aa
H2-T23
Fcer1g
Cd8b1
Psma2
Traf2
Vegfa
Kit
H2-Ab1
Xbp1
H2-D1
Cd63
Pdcd1lg2
Pecam1
Cd69
Stat5b
Plaur
Icam1
Ciita
Relb
Lcp1
Psmb8
Tapbp
Stat3
Icosl
Tnfaip3
Il2rg
Ccl3
Cxcl16
Cd274
Axl
Tnfrsf1b
Cd83
Lamp2
Ifnar1
Ifnar2
Ccl5
Il7r
Tgfbr2
Cd8a
Stat2
Zbp1
Tnf
Pou2f2
Tcf7
Irf5
Hck
Stat1
Isg20
Oasl1
Isg15
Xaf1
Kdr
Ebi3
Il1rl2
Tap1
Rsad2
Il1r1
Ifi35
Dll4
Ifit2
Irf4
Ccnd3
Syk
Klrd1
Card11
Cd180
Itgal
Notch1
Tnfrsf14
Atm
Cmah
Cxcr3
Mef2c
Il10ra
Klra17
Cd38
Ncf4
Ikzf1
Btk
Ifi27
Irf1
Ets1
Cd97
Sh2b2
Pik3cg
Amica1
Cd79b
Selplg
Ccr5
Cd244
Cd4
Itgb2
Cxcr4
Cfp

Supplementary Figure 10. The pDCs from mCAT and C57BL/6 mice show a comparable gene expression profile. pDCs purified from the spleens of C57BL/6 or mCAT mice were stimulated with 1 µg/mL of R848 during 9 hours. The RNA from pDCs cultured with either PBS or R848 was then extracted and the gene expression was analyzed using the nCounter® PanCancer ImmuneProfiling Panel kit from nCoulter (NanoString technology). After normalization, only the genes that were sufficiently expressed in the dataset (≥ 400 counts in total) were selected for the analysis. **(a)** Heatmap representation of the full list of expressed genes. Data are expressed on normalized gene counts. Samples and genes were sorted on the representation based on an unsupervised hierarchical clusterization. **(b)** Multidimensional Dimension Scalling (MDS) representation of the full list of the expressed genes. The variability of the gene expression profile between the samples is represented with a Euclidean distance in a 2 dimensional plans. Each spot corresponds to one single sample. **(c)** Heatmap representation of the differentially expressed genes. The genes selected had a q-value ≤ 0.05 (unpaired t-test with Benjamini Hochberg adjustment) when comparing C57BL/6 pDCs cultured with either PBS or R848, in addition to genes which had a q-value ≤ 0.05 when comparing mCAT pDCs cultured with either PBS or R848. Data are expressed on normalized gene counts. Samples and genes were sorted on the representation based on an unsupervised hierarchical clusterization.

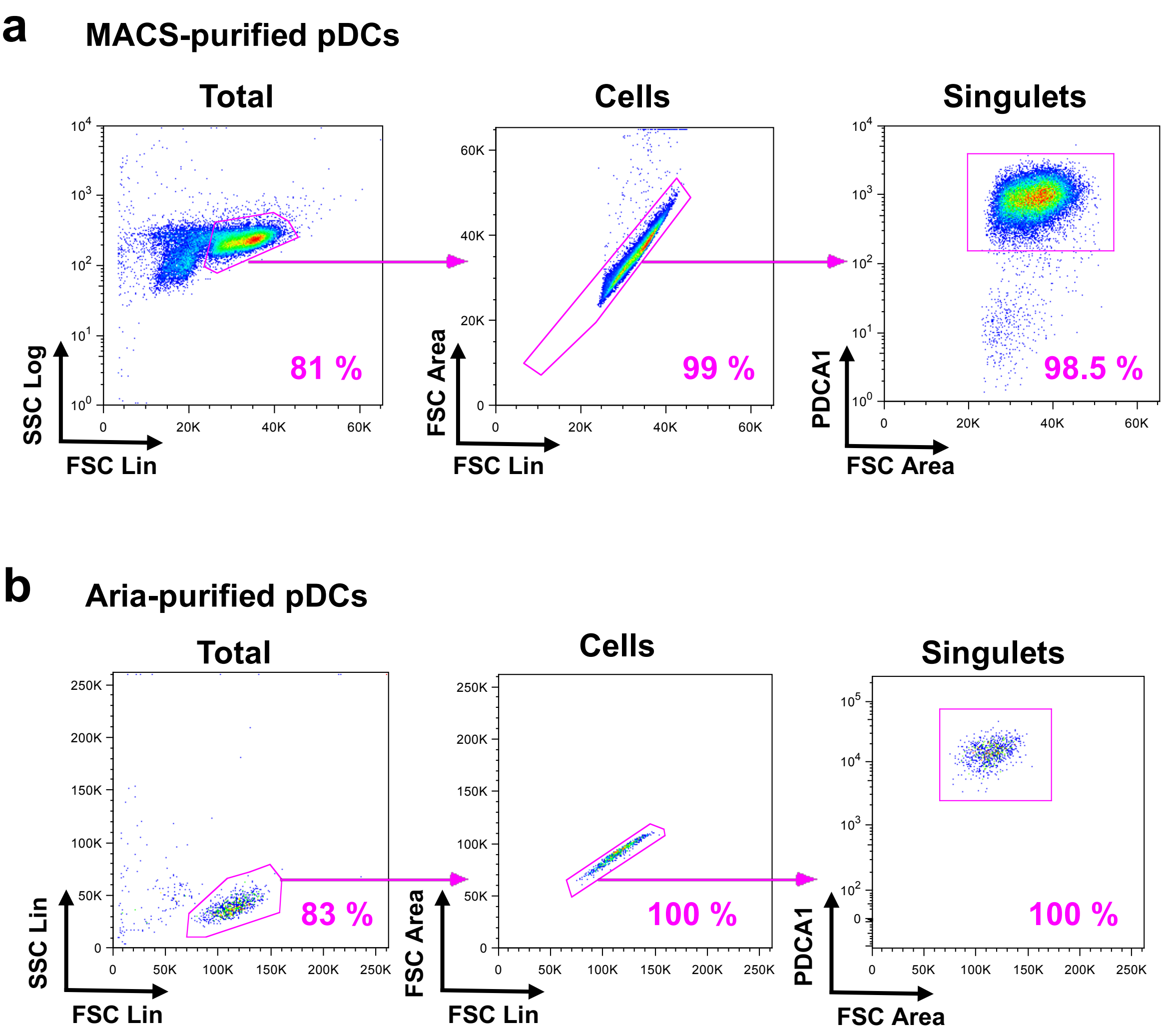

Supplementary Figure 11. Purification of pDCs. pDCs were purified from the spleens of C57BL/6 mice magnetically **(a)** or were FACS-sorted **(b)**. Numbers indicate the percentages of gated cells. The data are representative of more than 10 independent experiments.
